# Evaluating the Impact of Protocol-Driven Treatment for COVID-19 in an Emergency Department Observation Unit

**DOI:** 10.7759/cureus.29683

**Published:** 2022-09-28

**Authors:** Shehzad Muhamed, Jason Konzelmann, Laura Reed, Heather Holstein

**Affiliations:** 1 Emergency Medicine, Northeast Georgia Medical Center Gainesville, Gainesville, USA

**Keywords:** pandemic, emergency observation unit, covid-19, observation units, emergency department

## Abstract

Background

Hospital overcrowding and operating above capacity have occurred frequently throughout the COVID-19 pandemic. Both phenomena can lead to worsened patient outcomes; thus, it is imperative to find solutions that tackle both. Our goal was to create a treatment protocol for a subset of patients with mild to moderate COVID-19 infection that would combat inpatient overcrowding by diverting these patients to an emergency department (ED) observation unit (EDOU). This protocol was based on dynamic treatment guidelines and required regular updates to allow our team to provide the most up-to-date care throughout the pandemic.

Methods

This study is a retrospective chart review of all adult patients seen at two large suburban EDs for symptoms related to COVID-19 from April 2020 to January 2022. We subsequently identified adult patients who met the criteria for treatment with our COVID-19 protocol and were placed in our observation unit. These patients were identified using a flag for the protocol order set within our electronic medical record. Primary outcomes include the need for hospital admission, bounce back rate, and death rate.

Results

A total of 2,417 patients were treated in our ED observation units using our COVID-19 protocol. Our study population was evenly divided by gender, while a majority self-identified as white (76%). Five hundred two patients (20.8%) required admission to the hospital, and of these, 55 (11%) patients required intensive care unit (ICU) level of care. A total of 27 (1.1%) patients died. No deaths occurred for patients that remained within our ED observation units. Bounce back rates at the 48-hour, 72-hour, and seven-day marks were 3.6%, 4.6%, and 7.9%, respectively. Finally, we calculated a total of 284 inpatient days saved with the implementation of our protocol.

Conclusion

This study shows that our newly created protocol is effective in that it reduces the need for inpatient hospital admissions and results in low bounce back rates. Protocol-driven care in ED observation units can be a powerful tool against hospital overcrowding. Creating such protocols offers opportunities for hospital systems to provide efficient care at a significant cost savings without sacrificing quality of care. Our COVID-19 treatment protocol can be replicated by other hospital systems within their own ED observation units should any future similar outbreaks occur.

## Introduction

Emergency department (ED) observation units (EDOUs) have grown in popularity over the last two decades [1]. Multiple studies have demonstrated their benefits including shorter length of stays, lower costs, and higher patient satisfaction [2,3]. Decreasing length of stay and reducing costs have been crucial over the last 24 months given the COVID-19 pandemic [4-6]. Throughout the pandemic, emergency departments have been experiencing unprecedented levels of overcrowding and frequently operating above capacity. Hospital systems and emergency departments have been forced to find creative solutions to mitigate strain and free up valuable resources [7-9]. The utilization of EDOUs has been identified as a solution to provide efficient care [10].

Overcrowding is a well-known, multifactorial problem encountered by many health systems [11,12]. While initially causing volumes to fall during lockdown periods, the COVID-19 pandemic increased the frequency at which emergency departments suffered from overcrowding due to episodic surges [13]. Overcrowding leads to fewer practitioners following recommended treatment guidelines and ultimately worsens patient outcomes [14]. Thus, it is important that we recognize when this occurs and damper its effect.

At the onset of the pandemic, our emergency department group developed a protocol to place patients in our already functioning observation units to combat anticipated overcrowding within our inpatient units. A standard set of guidelines to place patients in observation does not exist, and only one study we found analyzes risk factors to guide disposition [15]. Frequently evolving treatment guidelines during the pandemic provided an additional challenge in making this protocol. However, our team designed a protocol based on best practices as supported in the literature and routinely updated it so we could provide the most advanced care. To date, we have placed over 2,000 patients in our observation units using our COVID-19 treatment protocol.

Protocol-driven treatment regimens can provide efficient care without sacrificing health outcomes. We hypothesize that a protocol designed to treat a subset of patients with mild to moderate COVID-19 infections had a positive impact on our health system by reducing the need for inpatient admission, maintaining low ED bounce back rates, and allowing us to reallocate resources for higher acuity patients.

## Materials and methods

This institutional review board (IRB)-approved study was a retrospective chart review of adult patients confirmed or suspected to have COVID-19 admitted to our ED observation unit. We included all patients over a 21-month period (from April 2020 to January 2022). Patients included were seen in two suburban EDs with a total annual combined volume of 150,000 patients. Our observation units have a combined capacity of 40 beds and a total annual volume of 15,000 patients. Outcomes included EDOU-to-hospital admission rate, bounce back rate, deaths, and inpatient days saved.

**Figure 1:**
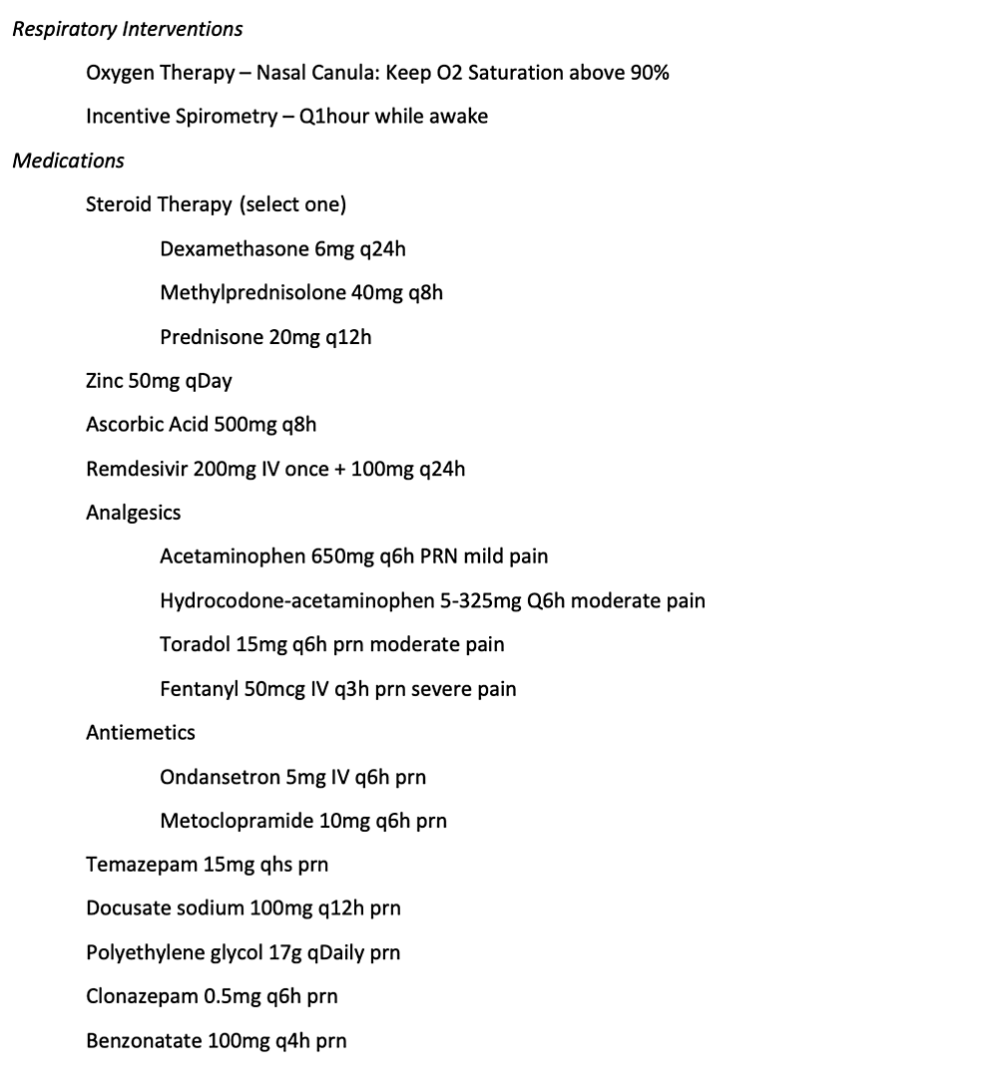
ED observation unit COVID-19 treatment protocol ED: emergency department

Approximately 21,000 patients were seen in our EDs for COVID-19-related symptoms during this time period. We identified patients who received treatment with our observation unit COVID-19 protocol (Figure 1) using a flag for the specific order set in our electronic medical record. All patients within our data set met the following criteria for admission to our ED observation unit: (1) suspected or confirmed COVID-19 infection within 14 days of illness onset and (2) oxygen saturation (SpO_2_) reading of less than 94% on room air on at least one occasion. Patients who required more than 4 L of oxygen to maintain oxygen saturation above 90% were not eligible for admission to the ED observation unit. All patients admitted to our ED observation units under any protocol must be able to ambulate on their own.

Data validation was performed on approximately 10% of our sample size. During this process, we identified seven patients that were initially treated on our COVID-19 protocol, subsequently admitted to the hospital, and eventually discharged. These patients returned to the emergency department within one week for readmission. These patients were removed from our data as this reflects inpatient care, not the efficacy of our protocol. Four other patients were excluded from the data set as they returned to our emergency department within one week for reasons unrelated to COVID-19. An additional 16 patients who left our emergency observation unit against medical advice were also excluded from the data set. Patients removed from the data set were individually reviewed by one investigator and then reviewed by other investigators in a blinded fashion. Only those patients with unanimous agreement were removed from the data set.

## Results

**Figure 2:**
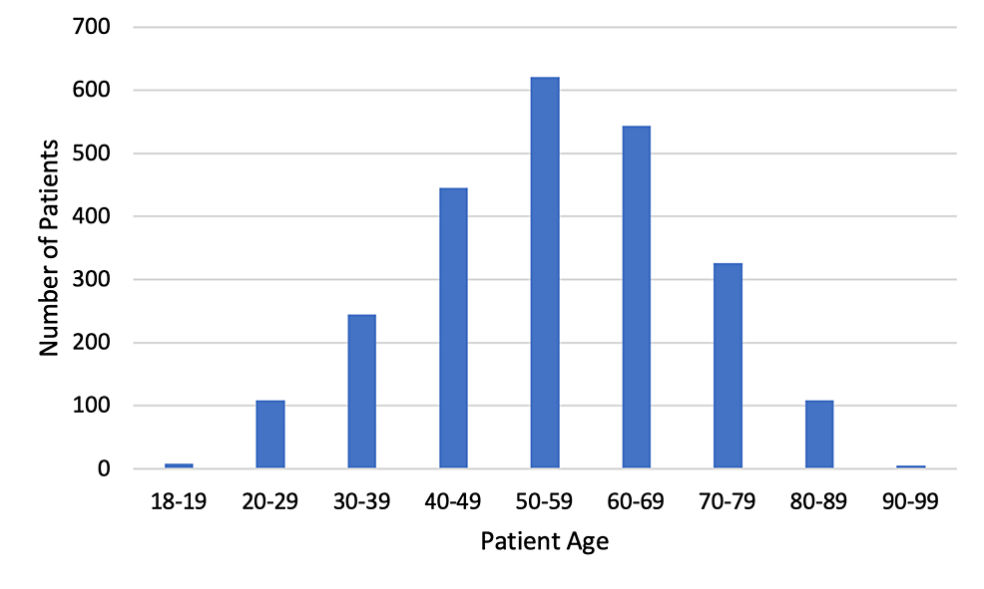
Age distribution of patients treated using the ED observation unit COVID-19 protocol ED: emergency department

**Figure 3:**
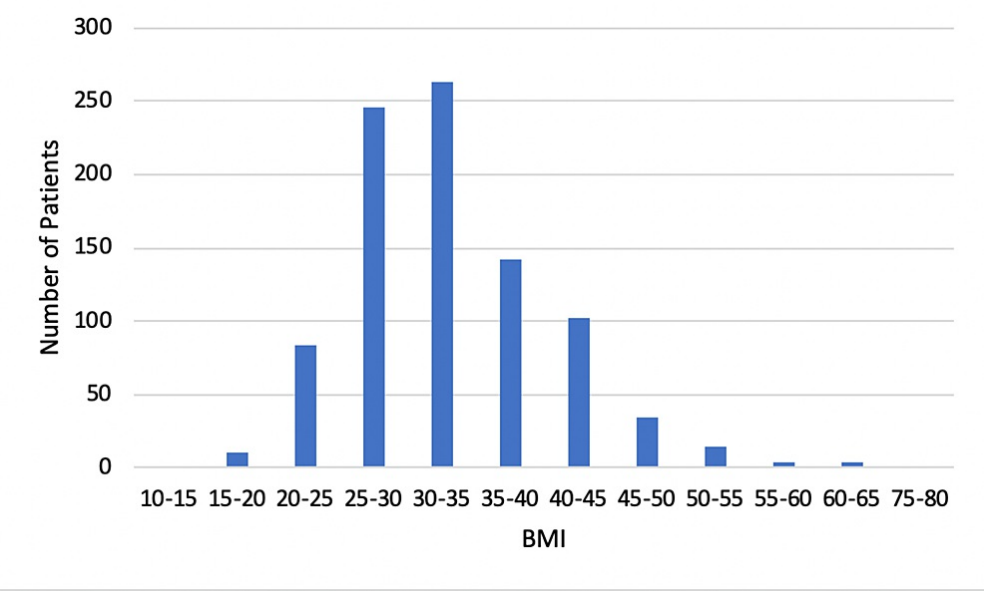
BMI distribution of patients treated using the ED observation unit COVID-19 protocol ED: emergency department

Over a 21-month period, 2,417 adult patients were placed in our ED observation units under the COVID-19 treatment protocol. Table 1 shows that a majority of our study population self-identified as white (76%). Patients identifying as Hispanic and black made up 15.8% and 8.3% of our study population, respectively. Patients were evenly divided by gender. The median age was 57 years old, and the median BMI was 32 (Figures 2-3). The two most common ED arrival methods were “walk-in” and “ambulance.” Primary chief complaints varied with “shortness of breath” as the most common.

**Table 1:** Characteristics of patients treated with the COVID-19 treatment protocol, n = 2,417

Median age (years)	57
Median BMI	32
Gender (%)	
Male	1,207 (49.9)
Female	1,205 (49.9)
Declined to answer	5 (0.2)
Race (%)	
White	1,833 (75.8)
Black	201 (8.3)
Asian	30 (1.2)
Others	353 (14.6)
Ethnicity (%)	
Not Hispanic or Latino	2,016 (83.4)
Hispanic/Latino	380 (15.7)
Others	21 (0.9)
Smoking status (%)	
Never smoker	1,525 (63.1)
Former smoker	623 (25.8)
Current everyday smoker	119 (4.9)
Unknown	150 (6.2)
Arrival method (%)	
Ambulance	466 (19.3)
Walk-in	1,798 (74.4)
Others	153 (6.3)

Of all patients, 502 (20.8%) required admission to the hospital as an escalation of their care. These patients had a median inpatient length of stay of four days. Fifty-five (11%) of those admitted required escalation to intensive care. Those patients who ultimately required intensive care unit (ICU) level of care had a median EDOU length of stay of 16.8 hours.

A total of 27 patients (1.1%) died. The median inpatient length of stay for the 27 patients who died was 23 days. The median age of patients who died was 67 years old. No patients who remained in the ED observation unit for the entirety of their care died. Table 2 provides additional information about the differences between patients who completed their care in our ED observation unit, patients that required inpatient admission, and patients needing ICU level of care.

**Table 2:** Characteristics of patients treated in ED observation alone, patients requiring inpatient admission, and patients requiring escalation of care from inpatient to ICU ED: emergency department; ICU: intensive care unit

	ED observation, n = 1915	Inpatient admission, n = 502	ICU, n = 55
Age (years), median	57	58	62
BMI, median	32	31.7	32
Gender (%)			
Male	958 (50)	252 (50.2)	30 (54.5)
Female	957 (50)	250 (49.8)	25 (45.5)
Race (%)			
White	1,453 (76)	380 (75.7)	45 (81.8)
Black	173 (9)	28 (5.6)	2 (3.6)
Asian	20 (1)	10 (2)	1 (1.8)
Others	269 (14)	84 (16.7)	7 (2.7)

About half of the patients admitted to the ED observation unit had an initial SpO_2_ between 95 and 100 (48.6%). Though this initial SpO_2_ does not meet the criteria for admission to the ED observation unit, this subset of patients had at least one subsequent documented SpO_2_ less than or equal to 94 during their ED course resulting in placement into the observation unit. The number of patients requiring hospital admission from the ED observation units generally increased as the documented initial SpO_2_ decreased (Table 3). Interestingly, patients with higher initial SpO_2_ most frequently returned to the ED for additional care.

**Table 3:** Initial SpO2 of patients treated with ED observation unit COVID-19 protocol, n = 2417 ED: emergency department; SpO_2_: oxygen saturation

SpO_2_ (%)	Number of patients on ED arrival	Admits to inpatient (%)	Revisits within 72 hours (%)	Revisits within seven days (%)
95-100	1,174	12.7	5.2	9.0
85-94	1,193	27.9	4.0	7.0
75-84	41	41.5	2.4	2.4
<75	9	22.2	0	0

A total of 1,915 patients were ultimately discharged from our ED observation units. The median length of stay for patients discharged from our observation units was 21.6 hours. A subset of patients did return at 48 hours (3.6%), 72 hours (4.6%), and seven days (7.9%) for additional care related to COVID-19. The two most common revisit triage complaints were “shortness of breath” and “coronavirus symptoms.” Across our study population, 284 inpatient days were saved.

## Discussion

At the onset of the COVID-19 pandemic, emergency departments throughout the country saw drastic decreases in volume. Over time, volumes returned to pre-pandemic levels along with several surges related to COVID-19. It is during these surge periods that hospital systems operate at their highest levels of strain, exhausting hospital resources. Our emergency department group anticipated that we may need a plan to address such a situation and created a COVID-19 treatment protocol to care for patients within our ED observation units. The purpose was to optimize ED flow by decreasing inpatient overcrowding and ultimately boarding in our emergency departments. This study showed the success we achieved in taking care of patients with mild to moderate COVID-19 infections.

To date, there is no such literature that provides a template for an ED observation unit COVID-19 treatment protocol as we do here. We designed a protocol with parameters to target a subset of patients who we felt could not be safely discharged from the ED and needed a short observation stay for additional care while determining if higher level of care was required. We feel our inclusion and exclusion criteria successfully identified patients that would do well after discharge as demonstrated by our low bounce back rates at 48 hours, 72 hours, and seven days (3.6%, 4.6%, and 7.9%).

All patients in our study population would have required inpatient admission or observation and occupied an inpatient bed had this protocol not been in place. Instead, we drastically decreased the rate of inpatient admission by discharging most patients with mild to moderate COVID-19 infection (79.2%). Our hospital system was able to free up valuable space for higher acuity patients presenting to us from our own emergency departments and as transfers from outside facilities. In our literature search, we found only one other similar study that admitted about 25% of its patients from an observation unit to inpatient [9].

One particular success of our protocol was the ability to quickly identify patients who initially presented with mild to moderate symptoms but required escalation of care. This is particularly impactful for those patients who ultimately needed ICU level of care. These patients had a median length of stay in our observation unit of 16.5 hours. This not only reflects the potential for rapid deterioration with SARS-CoV-2 but also validates our reason for creating this observation protocol.

Although we generally see our protocol as successful, an opportunity for improvement certainly exists. We observed two trends in our data that could be used to modify our inclusion and exclusion criteria. The first trend revealed a higher rate of admission in older patients compared to younger patients. Perhaps establishing specific age criteria would help us direct patients to a more appropriate level of care directly from the ED.

The second trend we noticed is those patients with a lower initial SpO_2_ upon presenting to the ED were more likely to require inpatient admission. Russell et al. found that hypoxia for patients being treated for COVID-19 in an observation unit was associated with the need for inpatient hospital admission [15]. Our protocol currently includes patients with at least one documented SpO_2_ below 94% and excludes patients requiring greater than 4 L of oxygen via nasal canula to maintain oxygen above 90%. Excluding patients based on initial SpO_2_ may be beneficial.

**Figure 4:**
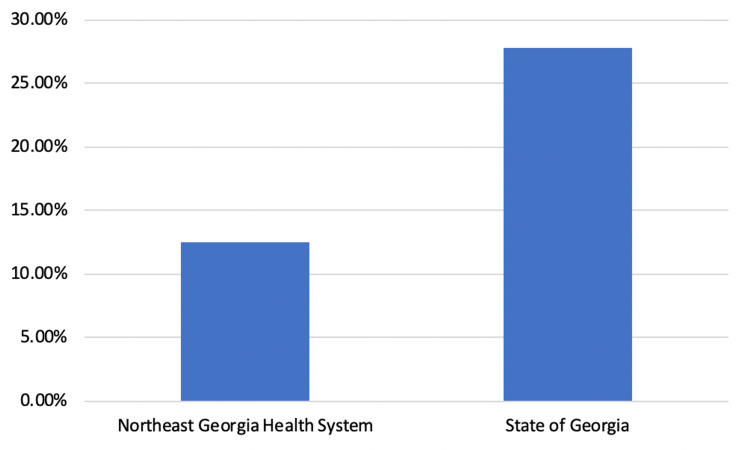
Percent of hospitalizations that have resulted in death due to COVID-19, from March 2020 to September 2021

The implementation of this protocol over 21 months saved 284 inpatient days. This presents a significant cost savings for our hospital system. Furthermore, it facilitates the ability to redirect resources. These benefits may be reflected in lower mortality rates seen in the county in which our system operates compared to other counties in Georgia (Figure 4). Other systems can reap similar benefits by providing care in ED observation units related to COVID-19, as well as other medical conditions.

## Conclusions

This study is not free of limitations. Primarily, it is a descriptive study and lacks a comparison group. Additionally, while we attempted to create a uniform treatment criterion, we cannot fully control for discrepancies in care between providers based on their individual care patterns. This is true for care that was provided in the ED, as well as the ED observation unit. A third limitation is that the study was conducted in a suburban setting with a predominantly white patient population, which may limit its generalizability. Lastly, this study was a retrospective study and presents evidence inferior to any similar prospective, randomized, control study.

This study shows that patients with mild to moderate COVID-19 illness can be successfully treated in an observation unit using protocol-driven care. Observation units can reduce the length of stay, reduce inpatient admissions, and free up valuable resources. Protocols can be created for a wide variety of conditions and be applied to situations that result in episodic volume surges. Future studies could examine the impact of modifying inclusion and exclusion criteria of our existing protocol and compare the efficacy of care in ED observation units versus traditional 23-hour observation.
